# Antitumor Mechanisms of Amino Acid Hydroxyurea Derivatives in the Metastatic Colon Cancer Model

**DOI:** 10.3390/ijms141223654

**Published:** 2013-12-04

**Authors:** Nina Šaban, Višnja Stepanić, Srđan Vučinić, Anita Horvatić, Mario Cindrić, Ivana Perković, Branka Zorc, Nada Oršolić, Mladen Mintas, Krešimir Pavelić, Sandra Kraljević Pavelić

**Affiliations:** 1 Rudjer Bošković Institute, Division of Molecular Medicine, Bijenička 54, Zagreb 10000, Croatia; E-Mails: nsaban@irb.hr (N.Š.); stepanic@irb.hr (V.S.); ahorvat@irb.hr (A.H.); mcindric@irb.hr (M.C.); 2 Croatian Academy of Sciences and Arts, Zrinski trg 11, Zagreb 10000, Croatia; E-Mail: svucinic@irb.hr; 3 Department of Medicinal Chemistry, University of Zagreb, Faculty of Pharmacy and Biochemistry, Antuna Kovačića 1, Zagreb 10000, Croatia; E-Mails: iperkovic@pharma.hr (I.P.); bzorc@pharma.hr (B.Z.); 4 Biology Department, Faculty of Science, University of Zagreb, Rooseveltov trg 6, Zagreb 10000, Croatia; E-Mail: nada.orsolic@biol.pmf.hr; 5 Department of Organic Chemistry, Faculty of Chemical Engineering and Technology, University of Zagreb, Marulićev trg 20, Zagreb 10000, Croatia; E-Mail: mmintas@fkit.hr; 6 Department of Biotechnology, University of Rijeka, Radmile Matejčić 2, Rijeka 51000, Croatia; E-Mail: pavelic@biotech.uniri.hr

**Keywords:** colorectal cancer, amino acid hydroxyurea derivatives, HDAC inhibition, anti-proliferative effect

## Abstract

The paper presents a detailed study of the biological effects of two amino acid hydroxyurea derivatives that showed selective antiproliferative effects *in vitro* on the growth of human tumor cell line SW620. Tested compounds induced cell cycle perturbations and apoptosis. Proteins were identified by proteomics analyses using two-dimensional gel electrophoresis coupled to mass spectrometry, which provided a complete insight into the most probable mechanism of action on the protein level. Molecular targets for tested compounds were analyzed by cheminformatics tools. Zinc-dependent histone deacetylases were identified as potential targets responsible for the observed antiproliferative effect.

## 1. Introduction

Colorectal cancer (CRC) is among the three most common malignancies worldwide, including breast and lung cancers. Late diagnosis, often accompanied by metastases, is a general problem for the treatment of this condition. Besides surgical intervention, the modern approach to CRC treatment strongly relies on the use of chemotherapeutics [1] and monoclonal antibodies [2]. Although combination and targeted therapy improved the therapy outcomes for CRC patients, high recurrence rates still pose a major lethality problem [3]. It is now accepted that a subpopulation(s) of malignant cells with stem cell properties may give rise to a hierarchy of proliferative and progressively differentiating cells and might account for invasiveness of tumors and decreased survival rates [4]. Current drugs do not target this particular subset of cells and novel therapeutic approaches, including novel drug entities, are thus interesting for advancements in CRC treatment.

**Figure 1.**
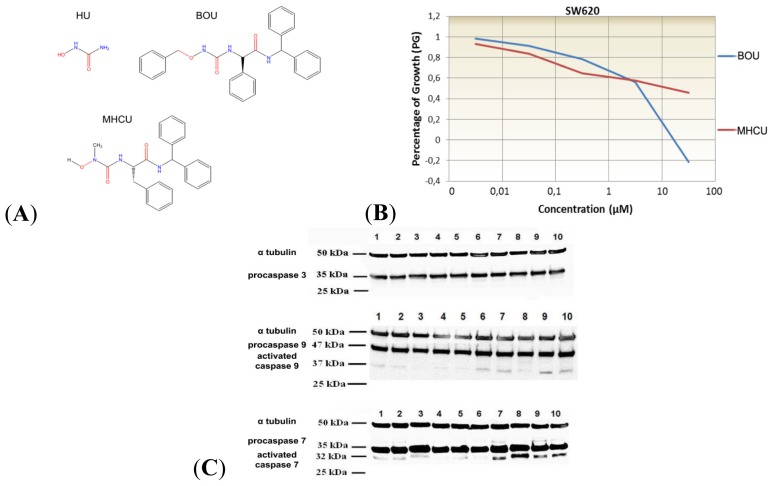
(**A**) Hydroxyurea (HU) and its derivatives *N*′-benzyloxycarbamoyl-d-phenylglycine benzhydrylamide (BOU) and *N*′-methyl-*N*′-hydroxycarbamoyl-l-phenylalanine benzhydrylamide (MHCU); (**B**) Concentration-dependent antiproliferative effect of BOU and MHCU on the SW620 cell line. Marginal means of survival were estimated as percentages of growth (PG); (**C**) Representative blots of SW620 cells treated with BOU and MHCU, probed with antibodies against human procaspase-3, procaspase-7 and procaspase-9. Treatments are as follows: **1**: control 24 h, **2**: BOU at 1 μM 24 h, **3**: BOU at 50 μM after 24 h, **4**: MHCU at 1 μM after 24 h, **5**: MHCU at 50 μM after 24 h, **6**: control after 72 h, **7**: BOU at 1 μM after 72 h, **8**: BOU at 50 μM after 72 h, **9**: MHCU at 1 μM after 72 h, **10**: MHCU at 50 μM after 72 h.

Hydroxyurea (HU) is a common antimetabolic cytostatic compound used to treat some types of cancer (Figure 1A) and a number of its derivatives exerting stronger antitumor potency and lower general cytotoxicity have been synthesized [5]. Similarly, Perkovic *et al*. [6] synthesized a series of novel l- and d-amino acid amide HU derivatives and evaluated their antiviral and cytostatic activity against malignant tumor cell lines, including leukemia and normal human fibroblasts [6]. In this paper, we report the biological mechanisms of action *in vitro*, *in silico* and *in vivo* of two compounds showing favorable, specific and concentration-dependent antiproliferative effects. The selected compounds, *N*′-benzyloxycarbamoyl-d-phenylglycine benzhydrylamide (BOU) and *N*′-methyl-*N*′-hydroxycarbamoyl-l-phenylalanine benzhydrylamide (MHCU), shown in Figure 1A, acted selectively on the colon tumor cell line SW620 in comparison with other tested tumor cell lines and normal human fibroblasts.

## 2. Results and Discussion

### 2.1. Amino Acid Hydroxyurea Derivatives BOU and MHCU Inhibit Proliferation of the Colon Cancer Cell Line SW620

We have previously shown that BOU and MHCU exerted the strongest antiproliferative effect upon a panel of tested cell lines, including the metastatic colon cancer cell line SW620 [6,7]. Tested compounds are both amino acid derivatives of HU, with the same amide moiety and a different amino acid part: BOU is a d-phenylglycine and MHCU is a l-phenylalanine derivative. In addition, they differ in the HU section: MHCU has a free hydroxy group, while the hydroxy group in BOU is protected by the benzyl residue. The presented study focused only on BOU and MHCU mechanistic analysis of metastatic SW620 cells, since metastases represent a major problem in cancer therapy. The antiproliferative assay results confirmed the previously observed concentration-dependent antiproliferative effects of these compounds on the growth of the SW620 cell line where BOU exerted a stronger cytotoxic effect while MHCU acted only as an antiproliferative agent (Figure 1B). The obtained IC_50_ values were 17.0 μM for BOU and 67.1 μM for MHCU.

### 2.2. Effects of BOU and MHCU on the Cell Cycle and Induction of Apoptosis

Tested compounds exerted weak cell cycle perturbations, but a strong cell death response, which was evidenced by a significant increase of the subG1 SW620 cell population (Table 1), indicative of apoptosis [8]. BOU caused a 31.2% increase in the subG1 phase after 24 h and a 7.8% increase after 72 h at higher concentration. MHCU induced only a 2.1% increase in the subG1 phase after 24 h and a 4.3% increase after 72 h at higher concentration. Interestingly, only BOU induced S phase arrest after 72 h at higher concentration.

Both compounds caused cell death evidenced by an increase of treated cells in the subG1 phase and by increased fraction of apoptotic cells (Table S1 in Supplementary Information). This effect was also stronger for BOU, probably partially attributable to its cytotoxicity. BOU caused a 6.8% increase of cells in the early stage of apoptosis after 24 h and a 9.2% increase of cells in the late stage of apoptosis/necrosis after 24 h in comparison with untreated cells. However, a lower rate of apoptosis induction was visible upon 72 h, which might be attributable to some other cell death mechanism, *i.e.*, senescence. MHCU increased the number of cells in the late stage of apoptosis at both tested concentrations after 24 and 72 h. Major apoptosis effectors, caspases 3 and 9, were expected to be activated by tested compounds since they are usually triggered in response to anticancer chemotherapy either through the extrinsic death receptor pathway [9] or the intrinsic mitochondria pathway [10]. Caspases 3 and 9, however, were not activated upon treatment of SW620 cells with BOU and MHCU (Figure 1C). Small amounts of cleaved/activated caspase 9 were detected in all treatments but were not significantly different in comparison with controls. Only caspase 7, tested due to a high degree of redundancy with caspase 3 and often activated instead of it [11], was cleaved/activated upon treatment with both compounds after 72 h.

The proportion of apoptotic cells assessed by the Annexin V assay was lower than the subG1 fraction measured by a flow cytometer. This might be explained by other cell death mechanisms occurring upon the treatment with tested compounds [12,13]. Indeed, only activation of caspase 7 was detected upon the treatment of cells with BOU and MHCU (Figure 1C), which may activate downstream targets in the absence of caspase 3 or act as a downstream effector for caspase-1-dependent pro-inflammatory effects or in the endoplasmic reticulum stress-induced death [11,14]. It has been shown that HDAC inhibitors (HDACi) might trigger oxidative stress [15] but reactive oxygen species (ROS) seem to be induced as an early post-treatment event rather than act as a persistent mechanism of action [16]. Even though a similar concentration-dependent antiproliferative pattern and caspase 7 activation (Figure 1B) were observed for both compounds, the cell cycle dynamics (Table 1) and percentage of apoptotic cell distribution (Table S1 in Supplementary Information) were somewhat different. The possibility of tested derivatives acting differently in the same cell line was thus studied in detail by global proteomic profiling, which proved successful in elucidating the molecular mechanisms triggered by novel antitumor compounds [17].

### 2.3. Protein Alterations in SW620 Cells Treated with BOU and MHCU

Global protein profiling of SW620 cells treated with BOU and MHCU showed significant alterations in qualitative and quantitative total proteome profiles after 24 and 72 h (Tables S2 and S3 in Supplementary Information). Identified differentially expressed proteins among control and treated cells are known to be involved in cell proliferation, apoptosis, regulation of the cell cycle, anti-inflammatory response, translation, transcription, structure of the cytoskeleton, oxidative stress, DNA repair, protein structure, tumor adhesion, invasion and metastasis as well as in cell metabolism.

Upon treatment of SW620 cells with BOU, a number of differentially expressed proteins were identified (Table S2 in Supplementary Information). The majority of identified proteins were down-regulated in BOU-treated cells, in particular, proteins driving metabolic processes, *i.e.*, hnRNP proteins that were previously identified as overexpressed in colorectal carcinoma [18]. Moreover, proliferation-driving proteins were down-regulated as well, *i.e.*, FUBP1, a known substrate of caspase 7 [19] that is probably degraded by caspase 7 in SW620 treated with BOU. Several other proteins that promote proliferation and/or block apoptosis were identified only in untreated SW620, *i.e.*, the main marker for proliferation PCNA [20]. It seems therefore that an immediate cell cycle arrest occurs upon treatment with BOU that ends up with cell death. These processes are connected to down-regulation of colorectal cancer malignancy biomarkers TAGLN2 and Gal-3 [21,22] as well as the metastatic phenotype marker PAI-RBP1 [23]. In conclusion, down-regulation of specific CRC proteins involved in tumor progression and invasion might explain the selective activity of BOU on the metastatic SW620 cell line in comparison with other tested tumor cell lines derived from solid tumors [6]. Moreover, it seems that cell death mechanisms might be triggered by metabolic changes and oxidative stress through activation of caspase 7 and down-regulation of two crucial oxidative stress-protecting proteins, DJ-1 and PRDX3, in treated SW620 cells (Table S2 in Supplementary Information).

A distinct protein pattern involving a number of inflammatory proteins was observed in SW620 cells upon MHCU treatment (Table S3 in Supplementary Information). For example, ANX1, which was up-regulated by MCHU treatment, has been already found to be an inducible endogenous inhibitor of NF-κB in human cancer cells and to provide a basis for novel molecular mechanisms of action for anti-inflammatory agents [24]. The possible MHCU anti-inflammatory mechanism was also evidenced by down-regulation of several proteins that drive inflammation, including HMG-1, PRDX2 and CRT. This result is interesting in the light of previous data showing anti-inflammatory effects on the systemic level of several HU derivatives [25].

Proteomic results showed that BOU increased oxidative stress while MHCU altered expression of proteins involved in the inflammatory response.

### 2.4. Docking of BOU and MHCU within HDAC Enzymes and the HDAC Inhibition Assay

**Figure 2.**
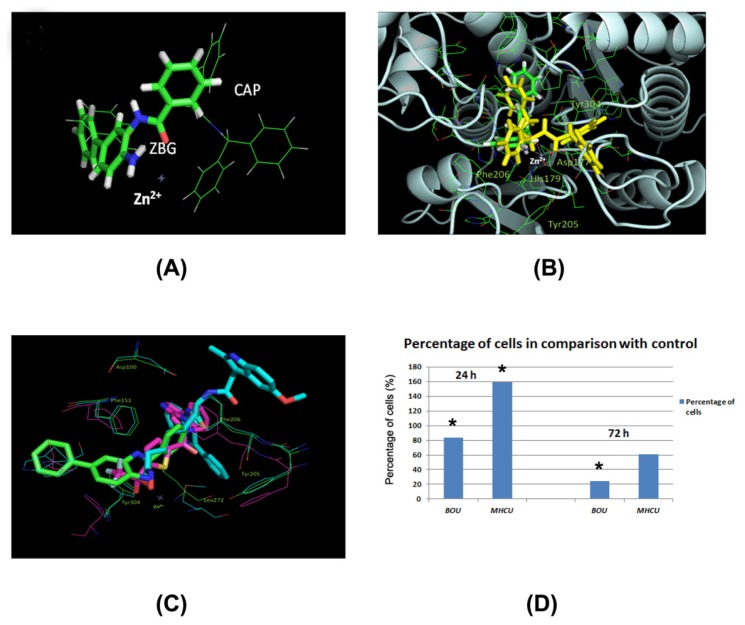
(**A**) The zinc-binding group (ZBG) and the CAP group of HDAC inhibitors; (**B**) Suggested binding mode of *N*′-benzyloxyureido derivative BOU (yellow) within the crystal structure 3MAX of HDAC2 complexed with the benzamide LXX (green); (**C**) Alignments of crystal structures of HDAC2 (3MAX, green), HDAC4 (2VQJ, pink) and HDAC8 (2V5X, cyan) complexed with the corresponding inhibitors; (**D**) HDAC enzymes (class I and/or II) activity in SW620 cells treated with BOU and MHCU for 24 and 72 h. Significant changes are marked with an asterisk (*).

A dozen substances similar to the studied compounds (Figure 1A) were found by PubChem search (similarity score ≥ 0.95). However, their biological effects were not described in scientific literature, with the exception of our previous report on cell growth inhibition activities [6]. Class I HDAC isoforms are highly expressed in colorectal carcinomas, particularly in the proliferating, de-differentiated tumors [26]. Hence, we tried to dock BOU and MHCU within deacetylase binding sites with solved X-ray structures. HU possesses metal-chelating and reducing properties, allowing compounds of this group to interact with a variety of metallo- and redox-active proteins [27]. The zinc-chelating enzymes that take part in cell proliferation are histone deacetylases (HDAC) of classes I and II [28]. Their most common competitive inhibitors are hydroxamate compounds. The common template of hydroxamate inhibitors of HDAC enzymes [29] contains a zinc-binding group (ZBG) and a CAP group interacting with surface residues of these proteins (Figure 2A) [30,31]. ZBG and CAP groups are usually connected by an extended linker containing hydrophobic fragments such as a phenyl ring. The hydroxamate group chelates Zn^2+^ in a bidentate fashion through its C=O and OH groups. According to the reported quantum-mechanical modeling, the potential isosteric ZBG for hydroxamate may be the HU group [30]. HU can be a satisfactory hydroxamate replacement if the rest of the molecule can compensate for the unfavorable desolvation effect on the binding affinity of hydrophilic HU [30]. This might be the case of lipophilic BOU (XlogP = 5.1) and MHCU (XlogP = 3.4) [32]. Higher lipophilic character of BOU may also account for its increased cellular uptake and thus more pronounced *in vitro* effects compared to MHCU. Herein, the binding site of the 3MAX structure of HDAC2 complexed with the inhibitor *N*-(4-aminobiphenyl-3-yl) benzamide (LLX) was used for BOU and MHCU docking (Figure 2B). This benzamide is more similar to the studied compounds than hydroxymates, most of which have unbranched alkyl linkers. While the Zn^2+^ ion, in the catalytic site of HDAC2, is coordinated by His141, His142, Asp177 and Asp265 [31], in the 3MAX complex it is coordinated by the ligand LXX and the residues Asp177, His179 and Asp265 demonstrating some kind of metal-binding flexibility (Figure 2B) [33]. Analogously, in the binding of BOU [6], the *N*′-hydroxyurea moiety should be oriented in such a way as to interact with the Zn^2+^ ion (Figure 2B). In that case, the benzyl substituent enters the lipophilic “foot” pocket adjacent to the catalytic place, while the freely rotating phenyl and biphenyl CAP groups can interact with the conserved lipophilic residues at the protein surface such as Tyr205 and Phe206 of HDAC2 (UniprotKB Q92769). Most amino acid residues around the binding site, particularly those at the protein surface, are positioned at the loops (Figure 2B), indicating that some degree of structural flexibility can be tolerated upon ligand binding. The other studied derivative, MHCU with an unsubstituted OH group (Figure 1A), has not shown significant inhibition of HDAC enzymes (Figure 2D), which indicates the importance of occupying the “foot” pocket and of hydrophobic interactions similarly to the case of LXX [33]. The previous observation that *O*-benzylhydroxyurea derivatives were generally more cytotoxic than their hydroxy pairs [6] is in accord with the HDAC inhibition activity of the studied derivatives. Stereochemistry has not been found to have a substantial impact on the cytostatic activity [6]. Similarly, it may be expected to be of minor significance for the inhibition of HDAC enzymes due to the flexible binding site. This assumption must be further confirmed by automated molecular docking and co-crystallization experiments.

Similarly as in HDAC 2, in HDAC 1 and 3 enzymes (based on protein sequence alignment since there are no crystal structures for these isoforms), there is also a “foot” pocket. However, in the HDAC4 and HDAC8 isoforms there are Pro (PDB 2VQJ) and Trp residues, respectively, at that place, preventing the use of ligands (Figure 2C). Thus, like benzamides, *O*-benzylhydroxyurea derivates can be inhibitors specific for HDACs 1–3 of class I [31].

*In silico* results pointed to HDACs of class I as potential molecular targets for compound BOU. The significant decrease of activity of HDAC of classes I and/or II, in the entire SW620 cell lysate treated with BOU at 50 μM concentration (Figure 2D), supported this finding. The HDAC enzymes of class I are overexpressed in CRC [26] and it has been reported that HDAC inhibitors might induce cell cycle arrest in SW620 cells in dependence on the inhibitor concentration [34]. We found that BOU induced cell cycle arrest in SW620 cells as well, suggesting that its inhibition of cancer cell growth might be mediated, at least in part, by arrest of the cell cycle progression caused by inhibition of HDAC of class I and/or II.

According to the *in silico* docking analysis, BOU probably inhibits class I HDACs 1–3 due to favorable occupancy of an available “foot” pocket near the zinc binding place by its *O*-benzyl substituent (Figure 2B). HDAC inhibitors (HDACi) are used clinically to treat malignancies because of their effects on apoptosis or cell death activation through anti-inflammatory effects caused by acetylation of non-histone proteins [35]. The effects of HDACi on inflammatory gene expression, however, may vary according to the cell type and the stimulus and might involve anti-inflammatory mechanisms such as down-regulation of IL-12, IL-1 and IL-6 [36] or induce a systemic pro-inflammatory effect [37]. Altered histone acetylation patterns have been reported in many cancers and HDACi may readily induce cell-cycle arrest or apoptosis although the exact cell death mechanism varies among different cells [38]. The mechanisms of BOU action might thus involve early oxidative stress, probably through induction of ROS, modulation of histone and non-histone acetylation pattern through inhibition of HDACs and cell death.

In contrast, the *in silico* docking analysis showed that interaction with the “foot” pocket near the zinc binding place of HDACs was not possible for MHCU. This result was substantiated by the HDAC colorimetric assay kit results as well (Figure 2D). HDAC assay demonstrated a stimulating activity of MHCU on the activity of HDAC enzymes. Induction of HDACs activity is in agreement with the altered regulation of several inflammatory proteins (Table S3 in Supplementary Information). It was already reported that anti-inflammatory effects of some drugs might be attributed to the activation of HDACs and specific acetylation/deacetylation patterns in cells [39,40] (ultimately leading to suppression of the inflammatory response).

The obtained information on the envisaged molecular interaction with cellular targets may provide a good basis for further optimization for improved amino acid hydroxyurea derivatives binding to HDACs and development of lead compounds.

### 2.5. *In Vivo* Activity of BOU

**Figure 3.**
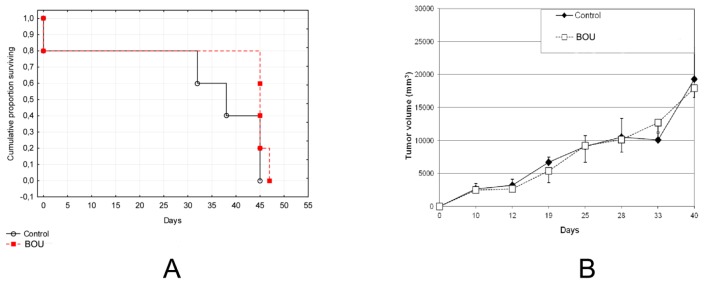
(**A**) Kaplan-Meyer survival graph for Balb/C mice inoculated intramuscularly with CT26WT tumor cells (1 × 10^6^ cells/mice) and treated with BOU at 1 mM/kg given intraperitoneally on days 1, 5, 10, 15 and 20. No statistical differences in overall survival growth of treated mice was observed in comparison with control mice (*p* = 0.1915; log-Rank test); (**B**) Tumor size in Balb/C mice inoculated intramuscularly with CT26WT cells (1 × 10^6^ cells/mice) and treated with BOU at 1 mM/kg given intraperitoneally on days 1, 5, 10, 15 and 20.

BOU exerted stronger antiproliferative effect compared to MHCU and was detected as a potential HDAC inhibitor. Therefore, its effect was evaluated *in vivo* on Balb/C mice inoculated with the colon carcinoma cell line CT26.WT. Rather high cytotoxicity observed *in vitro* and in the pilot experiment *in vivo* (data not shown) prompted us to diminish BOU dosages compared to the standard hydroxyurea doses used for studies in mice [41,42]. The mean survival time in the control group of Balb/C mice inoculated with the colon carcinoma cell line CT26.WT was 40 days, while it increased to 45.5 days in BOU; ILS % was 13.757% (data not shown). The overall survival period and tumor size after 45 days was not significantly different for mice treated with BOU (Figure 3). However, the treatment of animals showed a death reduction between 30 and 35 days upon treatment with BOU even though the tumor mass remained the same.

The absence of an overall effect on animal survival might be partially attributed to the low doses used for the *in vivo* experiments. This raises the question of toxicity and substantiates the need for further chemical optimization of BOU in relation to *in vivo* toxicity. Nevertheless, the therapeutic potential for BOU might be seen in combination with other small molecules with a complementary mechanism of action [43] or in chronic or autoimmune inflammatory disorders [44].

## 3. Experimental Section

### 3.1. Tested Compounds

Synthesis and antiproliferative effect of *N*′-benzyloxycarbamoyl-d-phenylglycine benzhydrylamide (BOU) and *N*′-methyl-*N*′-hydroxycarbamoyl-l-phenylalanine benzhydrylamide (MHCU) (Figure 1A) were described by Perković [6].

### 3.2. *In Vitro* Analyses

#### 3.2.1. Cell Culturing

The SW620 cells (colon carcinoma, metastasis) were purchased from American Type Culture Collection (ATCC, Manassas, VA, USA), cultured as monolayers and maintained in Dulbecco’s modified Eagle’s medium (DMEM) supplemented with 10% fetal bovine serum (FBS), 2 mM l-glutamine, 100 U/mL penicillin and 100 μg/mL streptomycin in a humidified atmosphere with 5% CO_2_ at 37 °C.

#### 3.2.2. Cell Viability Assay

Viability of cells was assessed by the MTT (3-(4,5-dimethylthiazol-2-yl)-2,5-diphenyltetrazolium bromide) assay. Briefly, the cells were plated into 96-well tissue culture plates (BD Bioscience, Franklin Lakes, NJ, USA) at a density of 3000 cells/well. After 24 h, BOU, MHCU and the solvent DMSO used for preparation of BOU and MHCU stock solutions were added into wells at freshly prepared 10-fold dilutions (0.01 to 100 μM) and incubated for 72 h. Absorbance was measured at 570 nm (ThermoLabsystems Multiskan EX, Beverly, MA, USA). Each point was performed in quadruplicate in three individual experiments. Measured absorbance values were transformed into cell percentage growth (PG) using the formulas proposed by NIH [45].

#### 3.2.3. Cell Cycle Analyses

A total of 2.5 × 10^5^ cells/well were seeded in 6-well plates (BD Bioscience, Franklin Lakes, NJ, USA). After 24 h, the cells were treated with BOU and MHCU at concentrations of 1 and 50 μM. After 24 and 72 h, the attached cells were trypsinized, combined with floating cells, washed with PBS and fixed with 70% ethanol. The cells were stained with 1 μg/mL of propidium iodide (PI) with addition of 0.2 μg/mL of RNAse A and analyzed using a Becton Dickinson FACScalibur flow cytometer (BD Bioscience, Franklin Lakes, NJ, USA) (20,000 counts). Each test point was performed in duplicate in three individual experiments. The results are shown as mean percentages from three separate experiments for each tested group. The percentage of cells in each cell cycle phase was based on the obtained DNA histograms and determined using WinMDI 2.9 software (freeware designed by Joe Troter, Purdue University, West Lafayette, IN, USA).

#### 3.2.4. Annexin Test for Detection and Quantification of Apoptosis

A total of 1 × 10^4^ SW620 cells were seeded in 8-well glass slides (Nalgene, Nunc Int., Rochester, NY, USA). After 24 h, the cells were treated with BOU and MHCU at concentrations of 1 and 50 μM. After 24 and 72 h, adherent cells were stained with Annexin-V-fluorescein labeling reagent (Boehringer Mannheim, Indianapolis, IN, USA) and propidium iodide (PI) according to the manufacturer’s recommendations. Assessment of apoptotic cells was performed under a fluorescent microscope (OLYMPUS, Center Valley, PA, USA) by manual counting. At least 100 cells were counted per sample. Annexin-V (green fluorescent) cells were found to be early apoptotic and Annexin-V and PI cells (red fluorescent) were found to be late apoptotic/necrotic. Percentage of apoptotic cells was expressed as the number of fluorescent cells compared to the total cell number.

#### 3.2.5. Western Blot Analysis

Untreated cells and cells treated with BOU and MHCU at concentrations of 1 and 50 μM, were lysed after 24 and 72 h in lysis buffer (50 mM HEPES, pH 7.5, 150 mM NaCl, 1 mM EDTA, 0.2 mM EGTA, 10% glycerol, 1% Triton X-100) and protease inhibitor cocktail (Roche, Basel, Switzerland). A total of 40 μg of proteins was resolved on 9% or 12% SDS-polyacrylamide gel at constant voltage (100 V) and subsequently transferred to a nitrocellulose membrane (BIO-RAD, Foster City, CA, USA) at constant electric current (200 mA) using a Mini-PROTEAN Cell (BIO-RAD, Foster City, CA, USA). Membranes were blocked with 4% non-fat dry milk in TBST (50 mM Tris base, 150 mM NaCl, 0.1% Tween 20, pH 7.5) and incubated overnight at 4 °C in 3% non-fat dry milk in TBST supplemented with primary antibodies against procaspase-3 (Santa Cruz Biotechnology, Dallas, TX, USA, diluted 1:250), procaspases 7 and 9 (monoclonal procaspase 7 and procaspase 9; Pharmingen BD, USA, diluted 1:200 and 1:1000, respectively), p21 (Pharmingen BD, Franklin Lakes, NJ, USA, diluted 1:200), p53 (Calbiochem, San Diego, CA, USA, diluted 1:150), and pJNK (Santa Cruz Biotechnology, Dallas, TX, USA, diluted 1:125). The membranes were then washed with TBST and incubated for 1 h at room temperature in TBST containing a secondary anti-mouse (GE Healthcare, Pittsburgh, PA, USA) or anti-rabbit (DakoCytomation, Glostrup, Denmark) antibody linked to horseradish peroxidase (diluted at 1:1000). The signal was visualized by the Western Lightning Chemiluminiscence Reagent Plus kit (PerkinElmer, Waltham, MS, USA) in the VersaDoc Imaging System 4000 (BIO-RAD, Foster City, CA, USA). Signal intensities of particular bands were measured and compared using Quantity One software (BIO-RAD, Foster City, CA, USA). Anti-β-tubulin (Sigma-Aldrich, Roedermark, Germany, monoclonal anti-β-tubulin mouse IgG, diluted 1:1000) was used as a loading control.

#### 3.2.6. Global Proteomic Profiling by 2D-Gel Electrophoresis and Mass Spectrometry

Untreated cells and cells treated with BOU or MHCU at a concentration of 50 μM were lysed after 24 and 72 h in 2-DE lysis buffer (7 M urea, 2 M thiourea, 4% CHAPS and 1% DTT) supplemented with 0.2% Bio-Lyte ampholyte, pH 3–10 (BIO-RAD, Foster City, CA, USA), nuclease mix (GE Healthcare, Pittsburgh, PA, USA) and protease inhibitor cocktail (Roche, Basel, Switzerland). 2D-gel electrophoresis analysis was performed according to Sedić [17]. Differential image analysis was performed using PDQuest software version 7.0 (BIO-RAD, Foster City, CA, USA) and total density in gel image was used as the normalization method. The following criteria were employed for the differential profiling of the proteins. From the gel, spots present only in treatments and spots resulting in 3-fold change in intensities between treatments and controls were excised manually for mass spectrometry analysis. Excised spots were destained using 10% acetic acid/40% methanol/50% water, *v*/*v*/*v*. After destaining, excised spots were washed three times with: (1) 50 mM ammonium bicarbonate buffer (pH 7.8), (2) 50% acetonitrile/50% 50 mM ammonium bicarbonate buffer (pH 7.8) (*v*/*v*) and (3) acetonitrile. The solvent was removed each time after washing. Finally, gel pieces were dried with a SpeedVac concentrator (Eppendorf, Hamburg, Germany) and protein digestion was performed by the use of trypsin (Merck, Munchen, Germany, 20 ng/mL) in 25 mM ammonium bicarbonate buffer (pH 7.8) overnight at 37 °C. Tryptic peptide solution was removed and dried with a SpeedVac concentrator (Eppendorf, Hamburg, Germany). Peptides were removed from the excised gel by adding 10 μL of 50% acetonitrile/5% trifluoroacetic acid (*v*/*v*), dried with a SpeedVac concentrator (Eppendorf, Hamburg, Germany), resuspended in 10 μL of 0.1% trifluoroacetic acid and purified using ZipTipC4 (Millipore, Billerica, MA, USA). The peptides were finally eluted with 10 μL of 80% acetonitrile/20% H_2_O/0.1% trifluoroacetic acid (*v*/*v*) and dried with a SpeedVac concentrator (Eppendorf, Hamburg, Germany). Tryptic peptides were resuspended in 5 μL of 5 mg/mL α-cyano-4-hydroxycinnamic acid (CHCA) in 50% acetonitrile/50% water (*v*/*v*), and spotted onto the MALDI plate.

Mass spectra were obtained on a MALDI-TOF/TOF mass spectrometer (4800 Plus MALDI TOF/TOF analyzer, Applied Biosystems Inc., Foster City, CA, USA). For each spot, 1600 shots per spectrum were taken with a mass range of 800–4000 Da, focus mass 2000 Da and delay time 500 ns. Trypsin autolysis peaks were used for internal calibration of mass spectra, providing mass accuracy within 21 ppm of their theoretical masses. Interpretation method was set to select the four most intense peaks (limit S/N 200) for tandem mass spectrometry (MS/MS) analysis, with exclusion of peaks generated from trypsin autolysis, matrix or acrylamide. MS/MS was achieved by 1 kV collision-induced dissociation (CID). Protein identities were established by applying the Global Protein Server (GPS) Explorer software version 3.6 (Applied Biosystems Inc., Foster City, CA, USA) against SwissProt, NBCI and MSDB using the Mascot package (Matrix Science, London, UK). Monoisotopic peptide masses were used for combined MS and MS/MS database searches with the following parameters: maximum allowed peptide mass error of 21 ppm, fragment mass tolerance ± 0.3 Da, minimum 5 S/N and one incomplete cleavage per peptide.

#### 3.2.7. HDAC Colorimetric Activity Assay Kit for Screening HDAC Inhibitory Compounds

HDAC Colorimetric Activity Assay Kit (BioVision, Milpitas, CA, USA) was used to measure HDAC activity in SW620 treated with BOU or MHCU at a concentration of 50 μM for 24 and 72 h and in untreated HeLa cells (positive control). Whole cell lysates were used for the analysis. All necessary reagents for the assessment of HDAC activity were supplied by the manufacturer. Briefly, HDACs remove acetyl from DNA substrates as well as artificial substrates containing acetylated groups. Deacetylation of the substrate sensitizes the substrate and subsequent treatment with the lysine developer within the kit produces a chromophore. Chromophore intensity was analyzed using an absorbance plate reader at 400 nm. Measured values were proportional to the HDAC activity and were presented as percentages in comparison with the controls. Each test point was measured in triplicate.

### 3.3. *In Vivo* Analyses

#### 3.3.1. Animals

The present study was approved by the ethical committee (Faculty of Science, University of Zagreb, Zagreb, Croatia). Male BALB/c inbred mice of the same sex, weighing 20–25 g, approximately 2 months old, obtained from the Department of Animal Physiology, Faculty of Science, University of Zagreb, were used. The animals were kept not more than 5 per cage under a 12 h reverse light/dark cycle with lights off at 6 pm and were maintained on a pellet diet (Standard Diet 4RF 21 GLP certificate, Mucedola, Italy) and water *ad libitum*. Animals were handled and weighed daily for a week to reduce any non-specific stress responses. Experimental groups comprised 8 mice each. The *in vivo* experimental protocol was modified according to the treatment of colon cancer recommended by the National Cancer Institute (NCI) [46]. Animal studies were performed in compliance with the guidelines in force in the Republic of Croatia (Law on the Welfare of Animals, Official Gazette #135, 2006; Regulations for the Environmental Conditions of Experimental Animals, Special Conditions for the Facilities and Experiment Categories, Official Gazette #176, 2004 and according to the Guide for the Care and Use of Laboratory Animals, DHHS Publ. (NIH) 86–123, 1985.

#### 3.3.2. Tumor Cell Line and Culture Conditions

The CT26 cell line is an *N*-nitroso-*N*-methylurethane induced undifferentiated adenocarcinoma of the colon, syngeneic with the BALB/c mouse [47,48]. For our studies, the CT26.WT cell line was grown in cell culture as monolayers in RPMI-1640 medium with 2 mM l-glutamine (Sigma Aldrich Chemie GmbH, Taufkirchen, Germany) supplemented with 10% fetal calf serum (FCS Gold, PAA Laboratories GmbH, Cölbe, Germany), 100 U/mL penicillin and 100 μg/mL streptomycin (PAA Laboratories GmbH, Cölbe, Germany). The cells were incubated at 37 °C in a humidified atmosphere containing 5% CO_2_. For the *in vivo* experiments, only CT26.WT cells of the first 3 serial passages after cryostorage were used. At the day of implantation, tumor cells were harvested from subconfluent cultures (70%–85%) by trypsinization (0.05% trypsin and 0.02% ethylendiamintetraessigsaure (EDTA), PAA Laboratories GmbH) and washed twice in a phosphate-buffered saline solution (PBS), and inoculated subcutaneously into BALB/c mice. Viability of cells was determined in a hemocytometer by observing the ability of intact cells to exclude Trypan blue dye and by phase contrast microscopy; it was found to be higher than 95%.

#### 3.3.3. Production of a Tumor in the Muscle Tissue of the Right Hind Leg

Tumor in the muscle tissue of the right hind leg was generated by sc injection of 1 × 10^6^ CT26.WT cells. Tumor was measured with a caliper every five days. Tumor volume was estimated from two-dimensional measurements: tumor volume (mm^3^) = (*a* × *b*^2^)/2, where *a* and *b* are the tumor length and width (mm), respectively [49,50].

#### 3.3.4. Survival Analysis

Animal life span was evaluated by surveillance of spontaneous death or by selective killing of animals showing signs of pain or suffering according to the established criteria. Results were expressed as percent of mean survival time of the treated animals over mean survival time of the control group (treated *vs.* control, *T*/*C*%). The percentage of increased lifespan (ILS%) was calculated by the formula: ILS% = (*T* − *C*)/*C* × 100, where *T* represents the mean survival time of treated animals and *C* the mean survival time of the control group. According to the criteria of the National Cancer Institute, *T*/*C* above 125% and ILS above 25% means that treatment had a significant antitumor effect [51].

#### 3.3.5. Statistics

Data were analyzed using the statistical software STATA 7.0 (Stata Press, College Station, TX, USA). Results were expressed as means ± S.D. Statistical significance was evaluated using ANOVA at *p-*value < 0.05 (normality of the distribution was assumed). Treatment-dose specific survival curves were calculated by the Kaplan-Meier method [52] and comparison between survival curves was made by the Mantel-Haenszel log-rank test (α = 5%) [53].

### 3.4. *In Silico* Analyses

Two approaches were exploited *in silico* for the study of potential biological macromolecular targets: (1) the similarity and sub-structure searches through open access compound databases PubChem [54] and ChEMBLdb [55]; and (2) isosteric replacement consideration, which is based on the fact that substitution of atoms or fragments, which have similar size, shape or electrical density results in creation of molecules of the same or similar biological activity. Molecular docking was employed to study BOU and MHCU potential as HDAC competitive inhibitors. For this purpose, crystal structures of HDAC enzymes from the Protein Data Bank (PDB) [56] were analyzed and the studied small molecules were docked manually in pre-selected structures using the PyMOL program (The PyMOL Molecular Graphics System, Version 1.0r1, Schrödinger, LLC, Portland, OR, USA). The PDB codes of analyzed crystal structures of HDAC enzymes of classes I and II were: 3MAX (HDAC2), 2VQJ (HDAC4), 3C0Z (HDAC7) and 2V5X (HDAC8). The compound equilibrium geometries were determined at the semi-empirical PM3 level by the ArgusLab 4.0.1 program (Mark A. Thompson, Planaria Software LLC, Seattle, WA, USA, http://www.arguslab.com) [57]. Lipophilicity coefficients XlogP were read off from PubChem compound summaries.

## 4. Conclusions

In conclusion, two amino acid HU derivatives, BOU and MHCU (Figure 1A), have similar phenotypic antiproliferative profiles [6] but elicit different biological response at the molecular level, as shown here by *in vitro*, *in silico* and *in vivo* analyses. *In vitro*, *O*-protected hydroxyurea derivative BOU showed somewhat stronger antiproliferative activity in comparison with *N*′-methyl-*N*′-hydroxy derivative MHCU on SW620 cells (Figure 1B). The cell-death mechanism observed for both tested compounds involves induction of apoptosis and probably oxidative stress that causes DNA damage for BOU (Table S2) and anti-inflammatory mechanisms for MHCU (Table S3). The benzyl substituted and free hydroxyl group of HU of BOU and MHCU, respectively, may account for their different biological response. This is illustrated by their inhibition of HDAC enzymes of classes I and II, as suggested by the *in silico* study and confirmed by *in vitro* testing (Figure 2). The observed HDAC inhibition activity of BOU points to the importance of occupying the “foot” pocket of class I HDAC enzymes and of hydrophobic interactions similarly to the case of benzamide inhibitors [33]. BOU exerted a significant short-term antitumor effect *in vivo* on the colon cancer mouse model (Figure 3A), which was attributed to alteration of inflammatory proteins and early oxidative stress mechanisms (Table S2) combined with HDAC inhibition.

## Supplementary Information



## Figures and Tables

**Table 1.** Flow cytometric analysis of SW620 cells treated with BOU and MHCU at concentrations of 1 and 50 μM for 24 and 72 h.

Treatment		Cell percentage (%) ± standard deviation			

		subG1	G1	S	G2/M
24 h	Control	5.4 ± 0.7	37.0 ± 0.5	45.6 ± 1.2	17.4 ± 0.9
	BOU, 1 μM	5.3 ± 0.6	34.6 ± 0.7 *	48.7 ± 1.4	16.7 ± 1.0
	BOU, 50 μM	36.6 ± 8.8 *	36.5 ± 0.7	47.3 ±2.4	16.2 ± 2.0
	MHCU, 1 μM	5.0 ± 0.7	35.9 ± 0.7	48.5 ± 0.9	15.5 ± 0.6
	MHCU, 50 μM	7.5 ± 0.3 *	35.6 ± 1.7	47.6 ± 1.5	16.8 ± 0.5

72 h	Control	5.5 ± 2.0	56.4 ± 1.7	28.5 ± 1.1	15.1 ± 1.5
	BOU, 1 μM	5.5 ± 2.0	57.2 ± 2.0	25.9 ± 1.3	15.4 ± 2.0
	BOU, 50 μM	13.3 ± 1.5 *	53.7 ± 2.3 *	34.2 ± 2.2 *	12.1 ± 0.8
	MHCU, 1 μM	4.3 ± 0.5	57.1 ± 2.7	29.7 ± 2.2	13.2 ± 1.7
	MHCU, 50 μM	9.8 ± 0.8 *	58.6 ± 1.6	24.5 ± 1.8	16.9 ± 1.1

* statistically significant.
