# Supplementary material for: Antitumor Mechanisms of Amino Acid Hydroxyurea Derivatives in the Metastatic Colon Cancer Model
Source: Int J Mol Sci. 2013 Dec 4;14(12):23654–71. doi: 10.3390/ijms141223654 (PMC3876069; doi:10.3390/ijms141223654)
Supplement: Supplementary file 1 [file ijms-14-23654-s001.pdf]

Supplementary Information

Table S1. Annexin-V test results for SW620 cells treated with BOU or MHCU at concentrations of 1 and 50 µM for 24 and 72 h.

		Number of cells (%)				
		**Control**	**BOU, 1 µM**	**BOU, 50 µM**	**MHCU, 1 µM**	**MHCU, 50 µM**
**24 h**	**Early apoptosis**	1.9	0.8	**8.7 ***	1.7	0.9
	**Late apoptosis/necrosis**	2.2	1.0	**11.4 ***	**5.5 ***	**7.8 ***
**72 h**	**Early apoptosis**	0.7	0.4	**2.4 ***	1.6	0.5
	**Late apoptosis/necrosis**	2.5	3.7	**6.4 ***	**6.2 ***	**8.7 ***

* statistically significant.

Table S2. Differentially expressed proteins in SW620 cells treated with BOU at a concentration of 50 µM for 24 and 72 h.

Protein	Abbreviation	Cellular process	Accession number	Expression level	Treatment (h)
Far upstream element binding protein 1 (FUSE binding protein 1) (DNA helicase V)	FUBP1	Proliferation	Q96AE4	Control	24/72
Plasminogen activator inhibitor 1 RNA- binding protein	PAI-RBP1	Tumor invasion and metastasis	Q8NC51	Control	24/72
Proliferating cell nuclear antigen	PCNA	DNA, synthesis, repair, cell cycle regulation	Q6FHF5	Control	24
Heterogeneous nuclear ribonucleoprotein B1-human	HNRPB1	Proliferation, repair	P22626	Control	24
DJ-1 protein (Oncogene DJ1)	PARK7	Sensor for oxidative stress, cell-growth	Q99497	Control	24/72
Transgelin 2	TAGLN2	Cytoskelet/ structural, CRC marker	P37802	Control	24
Phosphatidylethanolamine-binding protein	PEBP1	Antiapoptotic	P30086	Control	24
Transcription factor BTF3 (Basic transcription factor 3) (General transcription factor)	BTF3	Transcription and modulation of apoptosis	P20290	Control	24
Profilin 1 (Profilin I)	PFN1	Proliferation, migration	P07737	Control	24/72

Table S2. *Cont.*

Protein	Abbreviation	Cellular process	Accession number	Expression level	Treatment (h)
Chaperonin 10	HSPE1	Suppression of T-cell expression	P61604	Control	24
DnaK-type molecular chaperone-human	Hsp70	Antiapoptotic, proliferation	P11142	↓	24
Heterogeneous nuclear ribonucleoprotein L	hnRNP L	Regulation of transcription	P14866	Control	72
Stress-induced-phosphoprotein 1 (Hsc70/Hsp90-organizing protein) (Hop)	STIP1	Proliferation, antiapoptotic	P31948	Control	72
TAR DNA-binding protein-43	TDP-43	Transcriptional repression and mRNA and mRNA processing	Q13148	Control	72
Eukaryotic translation initiation factor 3 subunit 4	EIF3S4	Translation	O75821	Control	72
Poly(rC) binding protein 1	PCBP1	Antiapoptotic	Q15365	Control	72
GTP binding protein RanBP1	RANBP1	Mitosis	P43487	Control	72
Thioredoxin-dependent peroxide reductase, mitochondrial precursor (Peroxiredoxin 3)	PRDX3	Oxidative stress	P30048	Control	72
Phosphoglycerate mutase	PGAM	Glycolysis	P18669	Control	72
Eukaryotic translation initiation factor 4H	eIF-4H	Translation	Q15056	Control	72
Galectin-3 (Galactose-specific lectin 3 (Mac-2 antigen)	Gal-3	Proliferation, adhesiveness, apoptosis, CRC marker	P17931	Control	72
Nucleoside-diphosphate kinase, nm23-H1g	nm23	Phosphoryla-tion	P15531	Control	72
Translation initiation factor eIF-5A	eIF-5A	Translation	P63241	Control	72
Stathmin (Phosphoprotein p19) (Oncoprotein 18)	Op18	Proliferation	P16949	Control	72
Cofilin, non-muscle isoform (Cofilin-1) (18 kD phosphoprotein)	CFL1	Cell migration	P23528	Control	72
ENO1 protein, 2-phosphopyruvate-hydratase alpha-enolase	ENO1	Proliferation, motility, apoptosis, glycolysis	P06733	Control/↓	72
40S ribosomal protein S12	RPS12	Translation	P25398	Control	72
Triosephosphate isomerase	TIM	Glycolysis, metastasis	P60174	↓	72

Control – expressed only in untreated cells; ↓ – down-regulated in treated cells.

Table S3. Differentially expressed proteins in SW620 cells treated with MHCU at a concentration of 50 μM for 24 and 72 h.

Protein	Abbreviation	Cellular process	Accession no.	Expression level	Treatment (h)
Calreticulin	CRT	Chaperonin, regulation of Ca(2+), inflammation	P27797	Control	24
Immunophilin FKBP52 (FK506 binding protein 4)	FKBP52	Microtubule depolymerization	Q02790	↑	24
T-complex protein 1, alpha subunit	TCP-1-alpha	Protein folding, apoptosis	P17987	↑	24
Translation initiation factor eIF-5A	eIF-5A	Translation	P63241	↑	24
Fatty acid binding protein, epidermal (psoriasis associated)	E-FABP	Metabolism	Q01469	↑	24
Glyceraldehyde-3-phosphate dehydrogenase	GAPDH	Metabolism, glycolysis	P04406	↓	24
T-complex protein 1, gamma subunit	TCP-1-gamma	Protein folding, apoptosis	P49368	Treated	24
High mobility group protein 1	HMG-1	Transcription factors, tumor progression	P09429	Control	72
Ras-related protein Rab-11A, GTP-binding protein Rab 11	Rab-11A	Endosomal trafficking, cytokinesis	P62491	Control	72
FK506-binding protein 1A (Peptidyl-prolyl cis-trans isomerase)	FKBP1A	Protein folding, signal transduction, cell cycle regulation	P62942	Control	72
Ubiquitin	Ub	Cell cycle regulation, signal transduction, and stress response	P02248	Control	72
Annexin 1	ANX1	Anti-inflammatory, differentiation proliferation, apoptosis	Q05BR2	↑	72
Hydroxyacyl-Coenzyme A dehydrogenase, type II	HADH2	Metabolism	Q6IBS9	↓	72
Serine hydroxymethyltransferase mitochondrial precursor (Serine methylase)	SHMT2	Metabolism, proliferation	P34897	↓	72
Peroxiredoxin 2 (Thioredoxin peroxidase 1)	PRDX2	Oxidative stress	P32119	↓	72
Dihydrolipoyl dehydrogenase, mitochondrial precursor	DLD	Metabolism	P09622	Treated	72
Eukaryotic translation initiation factor 3 subunit 2 (TGF-beta receptor interacting protein 1)	TRIP-1 eIF3 beta	Cell signaling, ribosome assembly	Q13347	Treated	72

Control – identified only in untreated cells; Treated – identified only in treated cells; ↓ – down-regulated in treated cells; ↑ – up-regulated in treated cells.

© 2013 by the authors; licensee MDPI, Basel, Switzerland. This article is an open access article distributed under the terms and conditions of the Creative Commons Attribution license (http://creativecommons.org/licenses/by/3.0/).
